# Reduction in Acute Filariasis Morbidity during a Mass Drug Administration Trial to Eliminate Lymphatic Filariasis in Papua New Guinea

**DOI:** 10.1371/journal.pntd.0001241

**Published:** 2011-07-12

**Authors:** Daniel J. Tisch, Neal D. E. Alexander, Benson Kiniboro, Henry Dagoro, Peter M. Siba, Moses J. Bockarie, Michael P. Alpers, James W. Kazura

**Affiliations:** 1 Case Western Reserve University, Cleveland, Ohio, United States of America; 2 Papua New Guinea Institute of Medical Research, Goroka, Papua New Guinea; 3 London School of Hygiene and Tropical Medicine, London, United Kingdom; 4 Liverpool School of Tropical Medicine, Liverpool, United Kingdom; 5 Curtin University, Perth, Australia; National Institutes of Health, United States of America

## Abstract

**Background:**

Acute painful swelling of the extremities and scrotum are debilitating clinical manifestations of *Wuchereria bancrofti* infection. The ongoing global program to eliminate filariasis using mass drug administration is expected to decrease this and other forms of filarial morbidity in the future by preventing establishment of new infections as a consequence of eliminating transmission by the mosquito vector. We examined whether mass treatment with anti-filarial drugs has a more immediate health benefit by monitoring acute filariasis morbidity in Papua New Guinean communities that participated in a 5-year mass drug administration trial.

**Methodology/Principal Findings:**

Weekly active surveillance for acute filariasis morbidity defined by painful swelling of the extremities, scrotum and breast was performed 1 year before and each year after 4 annual mass administrations of anti-filarial drugs (16,480 person-years of observation). Acute morbidity events lasted <3 weeks in 92% of affected individuals and primarily involved the leg (74–79% of all annual events). The incidence for all communities considered together decreased from 0.39 per person-year in the pre-treatment year to 0.31, 0.15, 0.19 and 0.20 after each of 4 annual treatments (p<0.0001). Residents of communities with high pre-treatment transmission intensities (224–742 infective bites/person/year) experienced a greater reduction in acute morbidity (0.62 episodes per person-year pre-treatment vs. 0.30 in the 4^th^ post-treatment year) than residents of communities with moderate pre-treatment transmission intensities (24–167 infective bites/person/year; 0.28 episodes per person-year pre-treatment vs. 0.16 in the 4^th^ post-treatment year).

**Conclusions:**

Mass administration of anti-filarial drugs results in immediate health benefit by decreasing the incidence of acute attacks of leg and arm swelling in people with pre-existing infection. Reduction in acute filariasis morbidity parallels decreased transmission intensity, suggesting that continuing exposure to infective mosquitoes is involved in the pathogenesis of acute filariasis morbidity.

## Author Summary

The global program to eliminate lymphatic filariasis recommends 5 or more annual rounds of mass anti-filarial drug administration in order to reduce the reservoir of microfilaria to a level that will not sustain continuing transmission of infection by mosquitoes. We examined the incidence of acute filariasis morbidity defined by self-reported episodes of acute painful swelling of the extremities or scrotum in residents of communities in Papua New Guinea who participated in a 5-year mass drug administration trial. Acute morbidity events primarily affected the legs and arms, lasted less than 3 weeks, increased transiently following the first annual mass drug administration, and decreased progressively after repeated annual treatments. Risk factors for acute morbidity included pre-treatment microfilaremia and filarial antigenemia, pre-existing lymphatic pathology of the legs documented by physical examination, age older than 45 years and residence in communities where pre-treatment transmission intensity was relatively high. Results of our study indicate that mass administration of anti-filarial drugs has immediate clinical benefit to endemic communities in addition to future transmission cessation.

## Introduction

The goals of the ongoing global effort to eliminate lymphatic filariasis (LF) are to promote mass administration of anti-filarial drugs (MDA) in order to reduce and ultimately stop mosquito-borne transmission of *Wuchereria bancrofti* and to alleviate physical disability, suffering and economic disempowerment due to the pathology associated with this parasitic worm infection (www.filariasis.org). Increasing evidence supports the feasibility of achieving the first goal in circumstances where the public health infrastructure is capable of delivering MDA over a sufficient period of time and at the level of population coverage that is necessary to stop transmission. For example, in Egypt where pre-MDA microfilaria-positive infection rates were <5%, a national program consisting of annual treatment with diethylcarbamazine plus albendazole led to nearly complete elimination of microfilaria-positive carriers in the target population and possibly cessation of transmission when annual MDA was sustained for 5 years with population coverage >80% [1]. Results of MDA trials in areas where human and mosquito infection rates are higher than in Egypt also suggest that MDA may reduce the reservoir of microfilaria carriers sufficiently to prevent the establishment of new infections in children [2], [3]. By contrast, data pertinent to the impact of MDA on LF morbidity are more limited.

Developing, implementing and sustaining a public health program to treat and prevent LF morbidity are formidable tasks. Education in the use of footwear and attention to foot and leg hygiene aimed at reducing secondary bacterial infections of the skin have been shown to reduce the severity of chronic lymphedema and acute swelling of the leg [4]–[9]. However, the overall impact of this strategy, with or without coincidental MDA, has not been evaluated systematically for effectiveness or sustainability at the population level. This gap in knowledge is not surprising given the small proportion of LF-exposed individuals who experience clinically overt LF morbidity (usually <5% of infected individuals) and the logistical challenge of prospectively evaluating the three main forms of LF morbidity – chronic lymphedema of the extremities (e.g., elephantiasis), disfigurement of the scrotum (e.g., hydrocele) and acute filariasis morbidity characterized by transient painful swelling of the extremities (especially the leg) and, less commonly, the scrotum.

Fever with acute swelling and pain of an extremity or other anatomical locations such as the scrotum, here collectively referred to as acute filariasis morbidity (AFM), have been highlighted in the global elimination program as among the most common manifestations of LF morbidity that adversely affects individual and population health, particularly in communities where physical labor is important for economic well-being [10]–[12]. The pathogenesis of AFM is poorly understood, and is likely to have several etiologies that include the direct effects of filarial mediators on lymphatic function, immune-mediated inflammatory reactions to various stages of the *W. bancrofti* life cycle, innate immune responses to *Wolbachia* endosymbionts and secondary bacterial infections of the skin associated with pre-existing lymphatic dysfunction [8], [12]–[16]. Earlier studies have referred to acute painful swelling of the limbs in LF-infected individuals as acute filarial lymphangitis, posited to be due to inflammatory reaction elicited by dying lymphatic dwelling adult worms and/or allergic responses to third- or fourth-stage filarial larvae. A related syndrome with a prominent dermal component, referred to as acute dermatolymphangioadenitis, also occurs and is posited to be due to secondary bacterial infection of the skin [8]–[11]. These and other reports of AFM have focused primarily on the clinical management of self-referred patients. For example, prophylaxis with anti-filarial drugs and antibiotics given to patients in India who presented to local health facilities with fever and limb swelling decreased the rate of recurrence to one-sixth the pre-treatment rate [17]–[19]. On the other hand, prospective community-based studies of AFM are less commonly conducted and difficult to perform because the phenotype is transient (lasting from a few days to 1–3 weeks), unpredictable in the time of appearance, and confirmatory laboratory tests or biomarkers are non-existent. Previous attempts to identify risk factors for AFM have been limited to 1 year of observation in situations where MDA has not been implemented. These reports describe variability in the incidence of AFM according to endemic region, ranging from 0.03 episodes per person-year in Tanzania [20], 0.10 episodes per person-year in India [21] and Ghana [22], and 0.31 episodes per person-year in Papua New Guinea [23]. Increasing age and pre-existing chronic lymphatic pathology such as lymphedema of the leg were significant risk factors.

We report here the results of 5 years of weekly active surveillance for AFM in a population who participated in a community-randomized MDA trial conducted in Papua New Guinea from 1993 to 1998 [3], [23], [24]. The major objective of the trial was to compare the efficacy of single annual dose diethylcarbamazine alone to diethylcarbamazine combined with ivermectin in reducing transmission of *W. bancrofti* by the local mosquito vectors *Anopheles punctulatus* and *An. koliensis*. The two MDA regimens were observed to have similar efficacy in reducing LF transmission by the end of the 5-year surveillance period. Because this trial was conducted prior to current recommendations by the World Health Organization that educational programs to maintain skin and extremity hygiene be integrated into LF elimination programs [8], [9], [25], [26], we were able to determine whether MDA by itself affects the incidence and severity of AFM.

## Methods

### Ethics Statement

The Medical Research Advisory Committee of the Papua New Guinea Department of Health and the University Hospitals of Cleveland/Case Western Reserve University Institutional Review Board for Human Studies reviewed and approved conduct of the study. Written informed consent was obtained from participants older than 18 years and parents or guardians of persons whose age was 5 to 17 years. Children younger than 5 years and women who were pregnant at the time of the annual drug administration were not eligible to take anti-filarial drugs.

### Objectives

The objective was to determine whether the incidence of AFM was affected by annual mass administration of anti-filarial drugs that reduce the microfilaria reservoir and thereby decrease transmission of *W. bancrofti* infective third-stage larvae by local mosquito vectors. We tested the hypothesis that reduced mosquito transmission of *W. bancrofti* mediated by mass drug administration decreases acute filariasis morbidity as a consequence of diminished exposure to infective third- or fourth- stage larvae in the absence of curing pre-existing infection.

### Participants

All data were collected as part of a community randomized trial to test the efficacy of MDA with single annual dose diethylcarbamazine alone versus diethylcarbamazine plus ivermectin to reduce mosquito-borne transmission of *W. bancrofti*. The study was performed from 1993 to 1998 in the Dreikikir district of East Sepik Province, Papua New Guinea [3], [23], [27]. The observation period for AFM began 1 year before the first annual MDA and continued for each year after four annual MDAs.

### Description of Procedures

#### Prospective weekly monitoring for AFM

Field reporters recruited from local residents and trained by the research team performed weekly active surveillance in study villages by questioning each resident. The questionnaires asked whether a participant experienced swelling of the leg, arm, scrotum, or breast with or without fever on the day of the reporter's visit, during the preceding 1 or 2 days or during the previous week. Responses to questions were recorded on standardized printed morbidity forms in the local dialect. Data from the forms were entered into a database and linked with demography, census data, weekly migration records and records of anti-filarial drug administration, as described previously [23]. Demographic information such as age, sex and household of residence was updated monthly. AFM was defined as self-reported swelling of the leg, arm, scrotum or breast with the presence of fever during the previous week [23]. The duration of AFM was calculated such that a single event was defined by at least 2 weeks of negative questionnaire answers that preceded and followed 1 or more weeks of positive answers for AFM. Annual follow-up observations were standardized to the date of drug administration for each individual since study periods varied between annual MDA administrations for logistical reasons (intervals between consecutive annual MDAs ranged from 361 to 403 days per study year). The study population varied between 3,385 and 3,591 individuals for each year of observation. Person-time was calculated according to eligible weeks of observation for each individual. Time spent outside the study area and village of residence (e.g. due to migration) and prevalent disease time were not included in the observation time.

#### Annual determination of infection status and chronic LF pathology

Annual physical examination for signs of chronic LF morbidity such as lymphedema of an extremity and hydrocele were performed by JWK and other clinicians according to recommendations from the World Health Organization [28]. Yearly blood samples were obtained the night before administration of anti-filarial drugs in order to determine microfilaremia by the Nuclepore® filtration method. Og4C3 circulating filarial antigen levels were measured in samples obtained the day before the first MDA and 1 year after the fourth MDA. Filarial antigenemia was scored as negative, low or high as described previously [3], [24].

#### Entomological monitoring

Mosquitoes were captured by the landing catch method and results expressed as the annual infective biting rate, i.e. the number of blood-seeking mosquitoes containing at least 1 *W. bancrofti* infective larva that a person would be exposed to over a 1-year period [3], [24], [29].

### Statistical methods

Chi square tests were used to test differences in proportions. T-tests were used to compare differences in frequencies. Poisson regression (using the GENMOD procedure) was used to estimate the incident rate ratios (IRR) and 95% confidence intervals (CI) of AFM while incorporating multiple records of the same person in different years. Overdispersion was assessed using Pearson residuals. Negative binomial models were used to obtain standard error estimates when overdispersion was detected. Incidence ratios were calculated for baseline transmission intensity, age, sex, chronic disease status, microfilaremia status and density, and filarial antigenemia. Temporal effects of MDAs were evaluated by stratification and by including a variable for study year, controlling for repeated measures among individuals. In addition, stratified models were used to evaluate treatment-related effects after each MDA according to rapid (4 weeks immediately following MDA) and long-term (remaining 48 weeks of the study year) follow-up after MDA. Quasi-likelihood ratio tests were performed to compare models and select predictors for AFM incidence (i.e. age, sex, chronic LF pathology, presence or absence of microfilaremia, and filarial antigenemia). Model assumptions were evaluated by calculating deviance from the full model and autocorrelation plots of residuals. All analyses were performed with SAS version 9.1 (Carey, North Carolina, USA).

### Limitations

Observations in this study were made at a time (1993 to 1998) before the current World Health Organization recommendation for mass drug administration to eliminate lymphatic filariasis outside Africa be annual diethylcarbamazine combined with albendazole. We used diethylcarbamazine alone and diethylcarbamazine combined with ivermectin in our randomized community trial. These drugs were under consideration for mass drug administration at the time the study was performed. We do not believe that the use of these anti-filarial drugs diminishes the significance of the findings related to acute filariasis morbidity and MDA since: a) Mosquito transmission was effectively reduced in our earlier trial; b) There was no difference in the magnitude of transmission reduction effected by the diethylcarbamazine alone versus diethylcarbamazine combined with ivermectin; and, c) The anti-filarial drug with the highest activity against microfilaria and adult worms is diethylcarbamazine. Additional limitations relate to the fact that acute filariasis morbidity events that we tracked were self-reported and not documented by repeated physical examination or imaging studies of lymphatic vessels or lymph nodes.

## Results

Morbidity surveillance was performed on an open cohort during a 5-year annual MDA field trial, including 1 year of observation before the first annual round of MDA. Self-reported disease events and physical examinations for lymphedema of an extremity and hydrocele were observed for a total of 16,480 person-years. The absolute number of AFM events ranged from 1,445 during the pre-MDA year to 514 events during the year following 2 annual MDAs, corresponding respectively to incidences of 0.39 and 0.15 per person-year of observation (Table 1). The duration of AFM events was similar throughout the 5-year observation period, with 92% of events lasting 1 to ≤3 weeks as previously described in this study population [23]. Only 0.3% of events were recording as having a duration >10 weeks. AFM events were estimated for all potential disease locations: leg, arm, scrotum and breast. Leg events accounted for 76% of the total 4,149 AFM events (range of the 5 annual values 74–79%, Table 1). Events involving the arm occurred in 10% of the participants. All other locations accounted for <10% of AFM events, and occurred at rates of <0.02 per person-year of observation.

**Table 1:** Population characteristics and acute filariasis morbidity events according to study year.

	Study Year 1	Study Year 2	Study Year 3	Study Year 4	Study Year 5
	Pre-treatment Year	Year Following 1st MDA*	Year Following 2nd MDA	Year Following 3rd MDA	Year Following 4th MDA
Population (n)	3,385	3,390	3,382	3,437	3,591
Person-years of observation	3,742	3,050	3,336	3,104	3,248
Total number of acute events	1,445	944	514	601	645
Total number of leg episodes	1,075	710	404	446	498
All events/pyo†	0.39	0.31	0.15	0.19	0.20
Leg events only/pyo	0.29	0.23	0.12	0.14	0.15

*Mass drug administration (MDA) was conducted in Study Year 5 given but surveillance in the following year was not undertaken. †pyo = person-years of observation.

### Incidence of AFM according to timing of MDA

AFM incidence decreased from 0.39 per person-year of observation in the pre-treatment year to 0.31, 0.15, 0.19 and 0.20 after each of 4 consecutive MDAs (p<0.0001). The reductions in AFM rate following the first MDA according to disease location were 19%, 16%, 4% and 45% for leg, arm, scrotum and breast, respectively. The reductions in AFM rate following the second MDA were 58%, 67%, 60% and 77% of pre-MDA rates for leg, arm, scrotum and breast. Although the frequency of AFM decreased during the 5-year study period, the anatomical distribution of AFM events remained stable, with legs accounting for 74% of all events. AFM affecting the arm, scrotum and breast constituted the remaining 12%, 9% and 5%.

**Figure 1:**
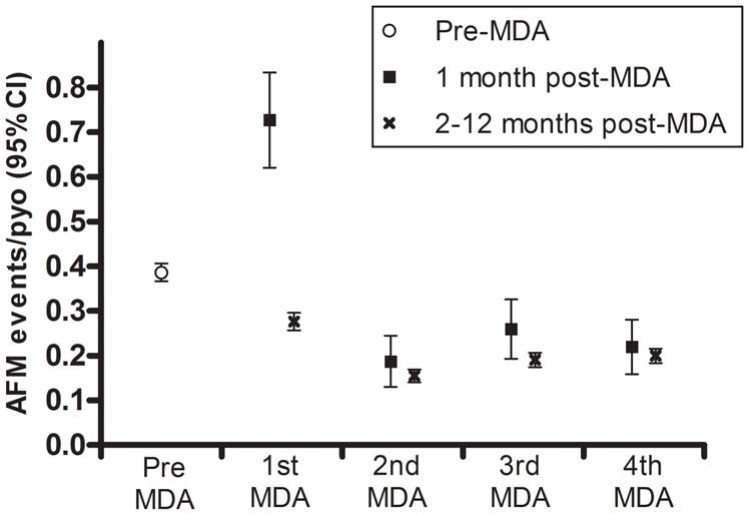
Incidence of acute filariasis morbidity during 5-year mass drug administration trial. Vertical bars around each value are 95% Confidence Intervals (CI). MDA = Mass Drug Administration (combined data from communities given diethylcarbamazine alone or diethylcarbamazine with ivermectin), AFM = acute filariasis morbidity, person-years of observation = pyo.

**Figure 2:**
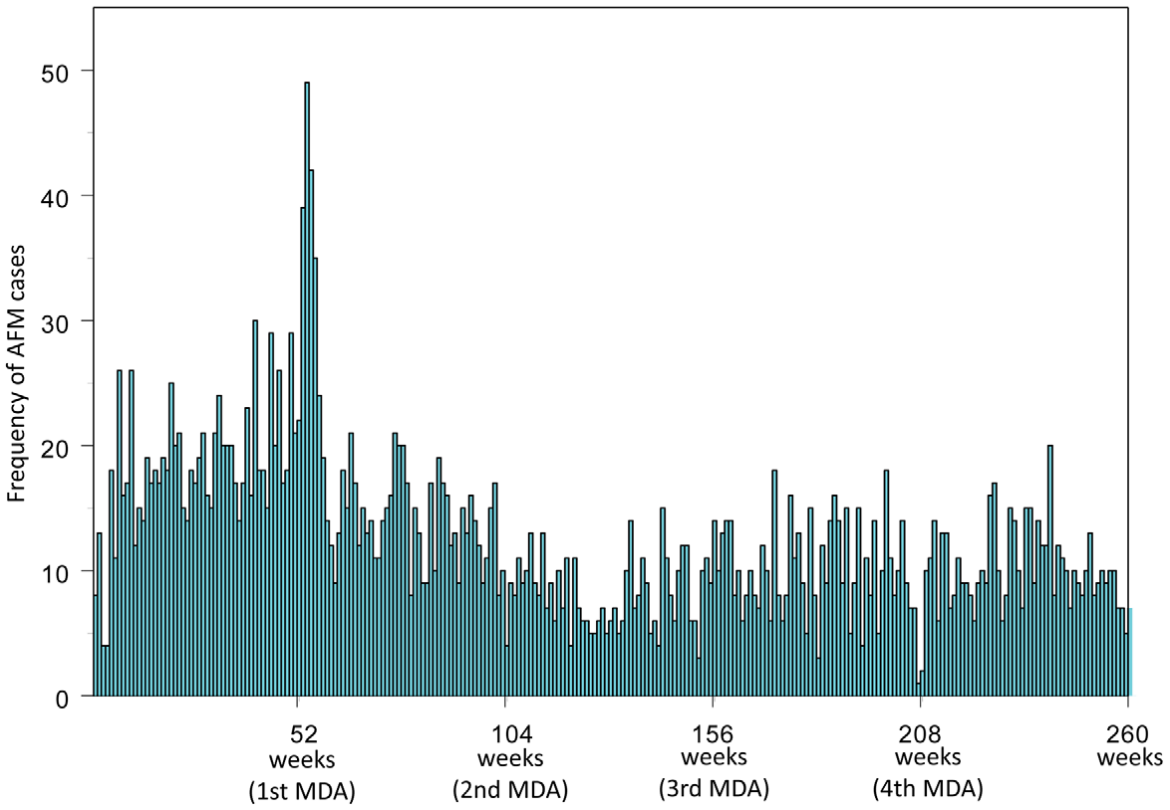
Weekly frequency of acute filariasis morbidity events during the 5-year observation period. Mass drug administration (MDA) was provided annually (i.e., at 52, 104, 156, 208 standardized weeks). The only peak of acute filariasis morbidity (AFM) occurred in the 4 weeks following the first MDA.

The annual incidence of AFM (all disease sites combined) decreased by 20% during the year following the first MDA and by 49–61% in subsequent years relative to pre-MDA rates (Table 1). In order to assess whether treatment-related acute events were putatively caused by rapid killing of microfilaria by anti-filarial drugs [30], AFM was evaluated separately during the first 4-week period after each MDA. The incidence during the 4-week period immediately following the first MDA was significantly higher than the remaining 48 weeks of the same year (0.73 versus 0.28 per person-year of observation, p<0.001) (Figure 1), and was the only time period during which weekly AFM events were more frequent than the pre-MDA year (227%, 115%, 73% and 10% increases over pre-MDA incidence for arm, scrotum, leg and breast, respectively). This large transient increase in AFM was not observed following subsequent MDAs (Figures 1 and 2) although a smaller increase did achieve statistical significance in study year 4 (0.26 per person-year of observation in the first four weeks after MDA versus 0.19 in the remaining 48 weeks of the same year, p = 0.023) (Figure 1).

### Microfilaremia as a risk factor for AFM

Microfilaremia was associated with the abrupt increase in AFM incidence during the 4-week period immediately following the first MDA; 8.3% of microfilaria-positive individuals experienced at least one episode of AFM versus 5.6% of microfilaria-negative individuals (p = 0.013). This corresponds to 35% greater risk for microfilaria-positive versus microfilaria-negative individuals during the month following the first MDA (95% CI = 1.14–1.61, p<0.0006). When the four week post-MDA period was excluded from the analysis of the study year, AFM incidence did not vary significantly according to microfilaremia. Furthermore, AFM rates did not differ according to microfilaremic status or density (categorized as 0, 1–9, 10–99 and ≥100 microfilaria per ml) during any other study periods.

### Transmission intensity and AFM

**Figure 3:**
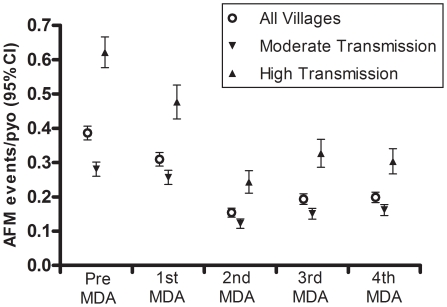
Incidence of acute filariasis morbidity events in moderate and high transmission villages. The open circles represent data from all 14 study villages combined. Villages are also stratified according to pre-MDA transmission intensity such that the down arrows represent moderate transmission villages (pre-MDA values for 8 villages were 24–167 infective bites per person per year) and up arrows represent high transmission villages (pre-MDA values for 6 villages were 224 to 742 infective bites per persons per year). High transmission villages experienced significantly greater AFM incidence in each year of the study compared to moderate transmission villages (p<0.001).

Study communities were stratified according to whether the pre-MDA transmission potential was moderate or high (24–167 versus 224–742 infective bites/person/year) [24]. AFM incidence during the pre-treatment year was significantly higher in the high-transmission communities (0.62 per person-year of observation, 95% CI = 0.58–0.67) than the moderate-transmission communities (0.28 per person-year of observation, 95% CI = 0.26–0.30, Table 2). AFM incidence after the second annual MDA was reduced by 57% relative to pre-MDA in areas of moderate transmission and by 61% in areas of high transmission, corresponding to a 97% and 84% reduction in transmission potential, respectively [24]. In the year after the fourth MDA the incidence was reduced by 43% in moderate-transmission communities and by 52% in high-transmission communities. The decreased frequency of AFM corresponded with a drop in annual infective biting rate to near zero in moderate-transmission communities and to 23–234 infective bites/person/year in the high-transmission communities 1 year after the fourth MDA [3]. The incidence of AFM in the high-transmission communities during the year after the fourth MDA (0.30 and 0.24 per person-year of observation for all anatomic sites combined and the leg only) was similar to the incidence of AFM among residents of moderate-transmission communities during the pre-MDA year. Accordingly, residents of high-transmission communities still experienced twice the risk of AFM compared to residents of moderate-transmission communities after 4 annual MDAs (Table 2, Figure 3).

**Table 2:** Acute filariasis morbidity incidence according to study year stratified by pre-treatment transmission intensity.

		Study Year 1	Study Year 2	Study Year 3	Study Year 4	Study Year 5
Transmission Index of Villages		Pre-treatment Year	Year Following 1st MDA*	Year Following 2nd MDA	Year Following 3rd MDA	Year Following 4th MDA
Moderate-Transmission	Population (n)	2,403	2,481	2,478	2,530	2,604
	Person-years of observation (pyo)	2,532	2,289	2,442	2,345	2,381
	Total number of acute events	711	587	298	354	385
	Total number of leg episodes	523	433	235	258	294
	Events/pyo†	0.28	0.26	0.12	0.15	0.16
	Leg episodes/pyo	0.21	0.19	0.10	0.11	0.12
High-Transmission	Population (n)	889	881	890	896	946
	Person-years of observation (pyo)	1,176	749	887	755	853
	Total number of acute events	731	357	216	247	259
	Total number of leg episodes	551	277	169	188	203
	Events/pyo	0.62	0.48	0.24	0.33	0.30
	Leg episodes/pyo	0.47	0.37	0.19	0.25	0.24

Moderate-transmission communities had infective biting rates of 24–167/person/year and high-transmission communities 224–742/person/year. *MDA = mass drug administration; †pyo = person-years of observation.

### Filarial antigenemia and its association with AFM

Filarial antigenemia was measured using plasma obtained 1 day before the first MDA (pre-MDA value) and 4 years later (1 year after the fourth MDA). The antigen-positive prevalence at these two time points (72% and 67%, respectively) did not significantly differ despite the intervening administration of four annual MDAs. Individuals who were filarial antigen-positive before MDA began were more likely to experience AFM than those who were antigen-negative (IRR = 1.47, 95% CI = 1.30–1.66, p<0.0001). Further, individuals with high filarial antigenemia were more likely to experience AFM events than those with low antigenemia (30% versus 16%, p<0.0001) or with negative antigen status (30% versus 14%, p<0.0001). 14% of individuals who were microfilaria-negative and antigen-negative (i.e. no detectable LF infection) during the pre-treatment year experienced events that were classified as AFM. During the final year of observation (following the fourth annual MDAs) 10% of individuals who were microfilaria-negative and antigen-negative (i.e. no detectable LF infection) experienced events that were classified as AFM. AFM events among those people without detectable infection (by antigen and microfilaremia in the first year and by microfilaremia only in subsequent years) corresponded to 6.7% of the total events observed over the 5 years of observation.

### Multivariable models

Multivariable models were used to evaluate independent risk factors for AFM. The variables included age (< versus ≥45 years), pre-treatment community transmission intensity (expressed as the annual infective biting rate), chronic LF pathology (leg lymphedema and hydrocele (documented during annual physical examination), microfilaremia, year of study, and the number of anti-filarial drug treatments actually taken (drug administration was directly observed). Filarial Og4C3 antigenemia was only included in models restricted to years in which this variable was measured (pre-MDA year and at the very end of year 5). With regard to the complete 5-year study period, individuals experienced the greatest risk of AFM in association with chronic leg lymphedema with a duration ≥1 year (IRR = 8.89, 95% CI = 7.00–11.30, p<0.0001; Table S1). Age and residence in a high-transmission community were also significant predictors of AFM (IRR = 2.20, 95% CI = 1.78–2.72, p<0.0001 and IRR = 1.58, 95% CI = 1.30–1.93, p<0.0001, respectively). Individuals who did not receive any MDA (e.g. refused, absent during the period of annual MDA), were at significantly greater risk for AFM than those who received 2 to 4 annual doses of anti-filarial drugs (IRR = 3.07, 95% CI = 2.25–4.20, p<0.0001). There was no significant difference in the risk of AFM related to drug regimen (diethylcarbamazine or diethylcarbamazine combined with ivermectin). Microfilaremia was included in preliminary models but dropped from final models due to the absence of observable effect.

Additional models were analyzed for individual study years with a focus on the pre-MDA year and the last year of observation following the fourth MDA (when Og4C3 antigen data were available). Microfilaremia was an independent predictor of AFM only in the year following the first MDA (1.48, 95% CI = 1.23–1.78, p<0.0001) and was dropped from final models of other years due to absence of observable effect. Age ≥45 years, high pre-MDA transmission potential and chronic LF pathology remained significant positive predictors of AFM overall and during the pre-MDA and after the fourth MDA year (p-values from 0.0452 to <0.0001; Table S2). The pre-treatment risk of AFM due to the independent effects of age ≥45 years (IRR = 1.28, 95% CI = 1.07–1.53, p = 0.0063), residence in a village with high pre-MDA transmission intensity (IRR = 2.56, 95% CI = 2.19–3.00, p<0.0001) and antigenemia (IRR = 1.26, 95% CI = 1.01–1.57, p = 0.0358) remained similar after four rounds of MDA, although antigenemia did not achieve statistical significance after four rounds of MDA (IRR = 1.52, 95%CI = 0.93–2.49, p = 0.0933) (Table S2). Though this study population experienced a decrease in chronic lymphedema of the leg and hydrocele prevalence after four MDAs [3], the risk of AFM associated with these two chronic forms of clinical pathology increased from an IRR of 3.56 (95% CI = 3.02–4.19, p<0.0001) to 7.95 (95% CI = 5.34–11.79, p<0.0001) over the same period (p<0.0001). In other words, among those individuals with pre-existing chronic lymphatic pathology, AFM incidence increased from 1.81 per person-year of observation (95% CI = 1.66–1.98) in the pre-treatment year to 2.16 (95% CI = 1.81–2.59) in the year following the fourth annual MDA (p<0.0001). Conversely, AFM risk in individuals without pre-existing chronic lymphatic disease decreased from 0.28 per person-year of observation (95% CI = 0.26–0.31) in the pre-treatment year to 0.18 (95% CI = 0.16–0.20) in the year following the fourth annual MDA (p = 0.17). This effect was significant independently of village transmission despite the fact that the overall prevalence of chronic disease was more common in high versus moderate transmission villages (10.5% versus 3.4%, p<0.001 pretreatment and 5.6% versus 2.5% following the fourth MDA, p = 0.025).

In order to evaluate whether the frequency of AFM events was predictive of the development of chronic lymphatic pathology, we compared AFM incidence among age-matched groups of individuals who underwent two physical examinations during the pre-treatment year, i.e. 12 months and 1–2 days before the first MDA. Among those individuals who were newly diagnosed with chronic lymphatic pathology during this one-year interval, the risk of experiencing an AFM event at any anatomic site was 0.64 (95% CI = 0.59–0.68), whereas the risk among individuals who were found not to develop chronic disease was 0.38 (95% CI = 0.35–0.42). For those individuals who had pre-existing chronic disease (i.e., positive physical findings at 12 months and 1–2 days before the first MDA) the risk was 2.38 (95% CI = 2.14–2.63). Each of these groups was significantly different from each other (p<0.0001). The results were similar when the analysis was restricted to leg events only. Taken together, these data suggest the AFM events precede the development of chronic lymphatic pathology and once established, chronic pathology further increases the likelihood of repeated AFM events. We did not perform a similar analysis for subsequent study years since the overall incidence of AFM was reduced by MDA.

## Discussion

We describe a comprehensive active surveillance of AFM events during repeated MDAs in an area of Papua New Guinea where LF is highly endemic. The incidence of AFM in this population decreased to 57% of the pre-treatment level after 2 annual MDAs in areas of moderate transmission and by 61% in areas of high transmission. This rapid decrease in AFM incidence was sustained over the entire 5-year surveillance period, thereby highlighting the potential of MDA to alleviate this feature of LF morbidity. Of concern, however, is the doubling of AFM events during the 4-week period following the first MDA (Figures 1 and 2). This transient increase was not, however, observed following subsequent MDAs. In fact, the multivariable models demonstrated that microfilaremia predicted AFM incidence only during the year following the first MDA, but not during the pre-treatment or any other post-MDA year. Drug-induced adverse events have been previously recorded following treatment with anti-filarial drugs, particularly among individuals with high levels of microfilaremia [30], [31]. The significantly higher incidence of AFM events among residents of communities with high versus moderate transmission intensities (Table 2) is consistent with these observations. The absence of periodic increases in AFM with subsequent treatments indicates that the individual microfilarial densities may have decreased sufficiently following the first annual MDA to eliminate drug-induced AFM following subsequent MDAs. More generally, these observations suggest that treatment naïve populations are likely to be at increased risk of adverse events during the early phase of MDA programs.

With regard to the study objectives, we have demonstrated a significant decrease in AFM following mass administration of anti-filarial drugs and the resulting decrease in indices of transmission by the local mosquito vectors. Our results showed that the incidence density of AFM remained stable following the first two MDAs, though the rates remained twice that reported from other LF endemic regions such as Tanzania [20], India [21] and Ghana [22]. AFM events in the latter phases of our study (post MDA years 3 and 4), when village-specific microfilaria levels and mosquito transmission of infective larvae were reduced respectively by 89–98% and 84–97% relative to the pre-MDA values [24], may be attributed to active LF infections that were not cured by earlier MDAs, pre-existing chronic LF pathology, and/or continued exposure to infective larvae [9], [10], [12]. Alternatively, the high rate of persisting AFM compared with other endemic regions (where post-MDA AFM was not examined) may be due to increased sensitivity of our follow-up protocols or low specificity of the case definition. With respect to the latter consideration, we did not attempt to determine whether self-reported fever accompanied by painful swelling of an extremity was due to an injury that promoted secondary bacterial infection of the arm or leg (shoes or other footwear were not used by most study participants), and malaria is highly endemic in this region of Papua New Guinea. We believe that malaria is unlikely to be a cause of incorrect categorization given that malaria-associated fevers in this region occur primarily in children younger than 5 years [32], whereas AFM occurs in adults. Regardless, the case definition used here is comparable to that used in other settings where these and other co-morbidities are present [11], [20]–[22].

Our results strongly support the current global strategy to eliminate LF through the use of repeated annual MDAs [33]–[38]. At the same time, the observations highlight important questions pertaining to the pathogenesis of AFM. Currently, acute morbidity events that involve painful swelling of the extremities, especially the legs, are classified according to whether they are due to secondary bacterial infection versus worm-associated etiologies [9], [10], [12]. Our study design did not allow us to distinguish between bacterial and filarial etiologies of AFM. To do so would require clinical monitoring in the home, bacterial culture of affected tissues, provision of antibiotics, and an estimate of adult worm death (e.g. scrotal ultrasound) [8], [9], [11], [39]. Biopsy of inflamed tissue to assess adult worm viability or the presence of *W. bancrofti* third or fourth-stage larvae is not justifiable clinically or ethically. Unlike studies of secondary bacterial infections of the skin, which to date have been limited to self-referred patients with pre-existing chronic lymphedema of an extremity, our study followed an entire population during repeated annual MDAs and noted that, though AFM rates were significantly greater in participants with pre-existing chronic LF pathology documented by physical examination, AFM rates also remained high for individuals with no pre-existing chronic LF pathology (incidence 0.28 per person-year of observation before MDA and 0.18 after four annual MDAs). Thus, while secondary bacterial infections associated with chronic lymphatic pathology of the leg may have played a role in the pathogenesis of some cases of AFM observed here, inflammatory reactions to adult worm or filarial larvae were also likely to have contributed. Regardless, the 48% decrease in AFM events following five rounds of MDA occurred in the absence of foot hygiene or antibiotic interventions which have been shown in other studies to independently decrease AFM events by 30 to 60% [6], [40]. This study supports current efforts to integrate hygiene and MDA for LF elimination. Finally, cases occurring in individuals without detectable LF infection presumably represent individuals who experienced morbidity events that were truly unrelated to *W. bancrofti* infection and/or have pre-patent LF infections that are below the limits of detection with the assays used here. It is also possible that some immune individuals have filarial antigen in their tissues from previous, cured infection without detectable antigenemia at the time of sampling.

The data reported here suggest various ways that the current strategy to eliminate LF may be modified from an operational perspective. Given the dramatic rise in AFM events that occurred immediately following the first MDA, community education regarding this possibility of experiencing such events may be important to sustain compliance over repeated annual MDAs, especially in endemic regions where heavily infected individuals and populations exist. Increased follow-up and case management of AFM during the early phase of MDA implementation may also be warranted. Finally, if indeed exposure to infective third stage or subsequent larval stages that develop in the lymphatics of recently re-infected or newly infected humans are involved in the pathogenesis of AFM, intensified efforts to reduce mosquito transmission such as by stepped-up vector control may be warranted.

## Supporting Information

Table S1 Summary rates for acute filariasis morbidity events based on a multivariable Poisson model.
(DOC)

Table S2 Multivariable Poisson model for pre-treatment year and year after fourth annual mass drug administration.
(DOC)

Checklist S1 (DOC)
