# Supplementary material for: Reduction in Acute Filariasis Morbidity during a Mass Drug Administration Trial to Eliminate Lymphatic Filariasis in Papua New Guinea
Source: PLoS Negl Trop Dis. 2011 Jul 12;5(7):e1241. doi: 10.1371/journal.pntd.0001241 (PMC3134431; doi:10.1371/journal.pntd.0001241)
Supplement: Table S1 — Summary rates for acute filariasis morbidity events based on a multivariable Poisson model. (DOC) [file pntd.0001241.s001.doc]

|  | Unadjusted model | | | Adjusted model | | |
| --- | --- | --- | --- | --- | --- | --- |
| Risk Factor | Incidence Rate Ratio | 95% Confidence Interval | p-value | Incidence Rate Ratio | 95% Confidence Interval | p-value |
| Age ≥ 45 years | 3.27 | (2.79, 3.84) | <0.0001 | 2.20 | (1.78- 2.72) | <0.0001 |
| High-transmission community | 2.21 | (1.83, 2.68) | <0.0001 | 1.58 | (1.30-1.93) | <0.0001 |
| Chronic Disease ≥1 year | 11.96 | (10.06, 14.22) | <0.0001 | 8.89 | (7.00-11.30) | <0.0001 |
| Chronic Disease < 1 year | 4.99 | (3.94, 6.32) | <0.0001 | 4.07 | (3.02-5.49) | <0.0001 |
| MDAs* received (none versus ≥ 2) | 0.98 | (0.77, 1.26) | 0.8861 | 3.07 | (2.25-4.20) | <0.0001 |
| MDAs received (1 versus ≥2) | 0.66 | (0.5, 0.87) | 0.0026 | 1.34 | (0.86-2.07) | 0.1941 |

*MDA=mass drug administration
