# Supplementary material for: Reduction in Acute Filariasis Morbidity during a Mass Drug Administration Trial to Eliminate Lymphatic Filariasis in Papua New Guinea
Source: PLoS Negl Trop Dis. 2011 Jul 12;5(7):e1241. doi: 10.1371/journal.pntd.0001241 (PMC3134431; doi:10.1371/journal.pntd.0001241)
Supplement: Table S2 — Multivariable Poisson model for pre-treatment year and year after fourth annual mass drug administration. (DOC) [file pntd.0001241.s002.doc]

|  | Year 1  (year before 1^st^ MDA*) | | | Year 5  (year after 4^th^ MDA) | | |
| --- | --- | --- | --- | --- | --- | --- |
| Risk Factor | Incidence Rate Ratio | 95% Confidence Interval | p-value | Incidence Rate Ratio | 95% Confidence Interval | p-value |
| Age ≥45 years | 1.28 | (1.07-1.53) | 0.0063 | 1.86 | (1.33-2.61) | 0.0003 |
| High-transmission community | 2.56 | (2.19-3.00) | <0.0001 | 1.47 | (1.01-2.14) | 0.0452 |
| Chronic disease | 3.56 | (3.02-4.19) | <0.0001 | 7.95 | (5.34-11.79) | <0.0001 |
| Antigen status | 1.26 | (1.01-1.57) | 0.0358 | 1.52 | (0.93-2.49) | 0.0933 |

*MDA=mass drug administration
